# The effects of onion (*Allium cepa* L.) dried by different heat treatments on plasma lipid profile and fasting blood glucose level in diabetic rats

**Published:** 2020

**Authors:** Taha Gökmen Ülger, Funda Pınar Çakiroglu

**Affiliations:** 1 *Ankara University Graduate School of Health Sciences, 06080 Ankara, Turkey*; 2 *Ankara University Faculty of Health Sciences, 06080 Ankara, Turkey*

**Keywords:** Allium cepa, Dyslipidemia, Hyperglycemia, Herbal medicine, Streptozocin

## Abstract

**Objective:**

This study aims to evaluate the effects of onion (*Allium cepa* L.) against hyperglycaemia and dyslipidemia and determine possible changes in these effects due to different heat treatments applied to onion.

**Materials and Methods:**

32 male Wistar-albino rats were divided into 4 groups as follows: the groups C and DC were fed with standard rat diet; the DLO group was fed with rat diet including 5% onion powder dried at -76°C in a lyophilizator, and the DFO group was fed with rat diet including 5% onion powder dried at 80°C in a furnace. Diabetes was induced in DC, DLO and DFO groups by injection of streptozotocin (45 mg/kg).

**Results:**

A decreasing tendency was observed in fasting blood glucose (FBG) values of DLO group during the experiment period and it was found that the 6th and 8th weeks values were significantly lower than the 1st and 2nd weeks values (p<0.05). On the other hand, no statistical difference was observed in the FBG values measured at different weeks in the DFO group. Significant differences were also observed among the groups in terms of plasma lipid values. DLO group was determined to have lower levels of triglyceride (p<0.001), LDL cholesterol and total cholesterol and higher levels of HDL cholesterol (p<0.05 for all cases) compared to the DC group whereas no significant difference in these values was found between the DFO and DC groups.

**Conclusion:**

Lyophilized onion powder may be protective against hyperglycaemia and dyslipidemia arising from diabetes. However, the heat treatments applied to onion affect this protective role negatively.

## Introduction

Diabetes mellitus (DM) is a chronic metabolic disease characterized by hyperglycemia accompanied by greater or lesser impairment in the metabolism of carbohydrates, lipids and proteins resulting from defects in insulin secretion, insulin action, or both (ADA, 2014 ▶). Due to increases in obesity, sedentary lifestyle and unhealthy nutritional habits, DM prevalence has increased worldwide. It is estimated that the number of people with DM, which is 425 million according to current data, will reach 629 million in 2045 (IDF, 2017 ▶). Nutrition habits have an important place in preventing and controlling DM which causes high health expenditures due to its acute (hypoglycemia, hyperglycemic hyperosmolar non-ketotic syndrome, diabetic ketoacidosis and lactic acidosis) and chronic (retinopathy, nephropathy, coronary heart disease, peripheral vascular disease and cerebrovascular diseases) complications. Although insulin therapy for Type 1 DM has no alternative, alternative treatment methods for treatment and care of Type 2 DM disease have become important because oral antidiabetic drugs (thiazolidinediones, sulfonylureas, and alpha-glucosidase inhibitors) cause undesirable side effects (Kodikonda and Naik, 2017 ▶). In this regard, more than 400 plant-derived products and more than 120 naturally-derived products are currently used to provide support in treatment of diabetic patients (Karaman and Cebe, 2016 ▶).

Onion from the Allium vegetables, is one of the foods thought to have a protective role against DM due to its contents of flavonoids (quercetin) and organosulfur compounds such as s-allyl cysteine sulfoxide, s-methyl cysteine sulfoxide, diallyl trisulfide, dimethyl trisulfide, propenyl propyl disulfide, dipropyl disulfide, propenyl methyl disulfide, methyl propyl trisulfide and dipropyl trisulfite (Eldin et al., 2010 ▶; Islam and Choi, 2008 ▶). However, although many studies reported that onion has hypoglycemic (Ikechukwu and Ifeanyi, 2016 ▶), fibrinolytic (Torres-Urrutia et al., 2011 ▶), antihyperlipidemic (Bang et al., 2009 ▶), antithrombotic (Yamada et al., 2004 ▶), antioxidant (Yamamoto et al., 2005 ▶), and antibacterial (Kabrah et al., 2016 ▶) effects, there are no *in-vivo* studies conducted to investigate possible changes in the functional activity of the onion subjected to heat treatment. Studies on the effects of different cooking methods and different heat treatments on onion’s flavonoid and ascorbic acid contents, reported that flavonoid and ascorbic acid contents decreased due to increased temperatures (Crozier et al., 1997 ▶; Ewald et al., 1999 ▶; Sharma et al., 2015 ▶). 

Onion is one of the most widely used vegetables in the world cuisine and is generally consumed after high heat treatment. In this regard, this study aimed to compare functional efficiency of the onions exposed to different heat treatments, against diabetes.

## Materials and Methods

**Animals and experimental induction of diabetes**

This study was approved by the Animal Experiments Local Ethics Committee at Ankara University (Meeting No. 2017-3, Decision No. 2017-3-21). This study was financially supported by the Scientific Research Projects Unit at Ankara University (Project No. 17L0241003). Thirty-two male Wistar-albino rats aged 3-4 months, weighing 280-330 g were obtained from the Experimental Animals and Research Laboratory at Ankara University and used in the study. The animals were housed in the cages under standard conditions (12:12 hr light dark cycle, 50% relative humidity at 21°C). After the adaptation period, the rats were randomly divided into 4 groups of 8 rats in each group. Then, streptozotocin (STZ, Sigma-Aldrich, USA) at a dose of 45 mg/kg body weight in 0.1 M citrate buffer (pH 4.5) was injected intraperitoneally (Indumathi et al., 2018 ▶) into 24 rats to induce experimental diabetes. Seven days after the injection, fasting blood glucose (FBG) measurements were done by drawing blood from their tail veins after 12 hr of fasting, using a glucometer (Accu-Chek Performa Nano, Turkey); accordingly, the rats with FBG values exceeding 250 mg/dl were considered diabetic. Then, for the following 8 weeks, the groups C (Control) and DC (Diabetic Control) were fed with standard rat diet, the DLO group (Diabetic + Lyophilized onion) was fed with rat diet including 5% onion powder (dried at -76°C in a lyophilizator), and the DFO (Diabetic + Furnaced onion) group was fed with rat diet including 5% onion powder (dried at +80°C in furnace). Feed and water consumptions of the groups were monitored daily, and FBG levels were measured once every 2 weeks. At the end of week 8, the rats were anesthetized after 12 hr of fasting, using an injection of 10 mg/kg xylazine and 70 mg/kg ketamine HCl; then, they were sacrificed taking 5-7 ml of intracardiac blood. The rat blood samples were centrifuged and the pre-determined parameters were checked.

**Preparation of onion powder and rat diets**

After separating the head and stem parts of the onions (*Allium cepa* L.), the shells were peeled off and diced using a ceramic knife. Some of the diced onions were firstly placed in a freezer (Operon, South Korea) and stored at -78°C for 24 hr. After storage, the onions were transferred to the lyophilizer and dried at -76°C under 200 mTorr engine pressure. The remaining diced onions were directly placed in a furnace (Vestel AFB-1004, Turkey) and dried at +80°C using its fruit-dry mode. Lyophilized and furnaced onion powders were added to standard rat chow at a dose of 5% separately and used for DLO (lyophilized onion) and DFO (furnaced onion) groups diet. Ingredients of the standard rat chow prepared according to National Research Council (NRC) standards, were mixed homogeneously by adding onion powders before pelleting and then, pelleted for animal consumption.

**Blood glucose and serum lipids**

Blood glucose was measured for all the animals in samples taken from the tail vein using an appropriate kit (Accu-Chek Performa Nano, Turkey). The concentrations of serum total cholesterol (TC), triglycerides (TG), high density lipoprotein cholesterol (HDL-C) and low density lipoprotein-cholesterol (LDL-C) were determined using a commercial kit (Rel Assay, Turkey). Biochemical assays were done in the Mega Medical Biochemistry Laboratory with the Rel Assay auto-analyzer.

**Statistical analysis**

Using Statistical Package for the Social Sciences (SPSS) version 18.0 (SPSS Inc., Chicago, IL, USA) software, the parameters except for FBG, were statistically analyzed by one way analysis of variance (ANOVA) with Tukey *post hoc* test. FBG values measured during the experiment were assessed using repeated-measures ANOVA with Bonferroni correction. Results are expressed as mean±SEM for groups of eight animals each. Statistical significance was considered at p<0.05.

## Results

**Body weight, and food and water intake**

No significant difference was found among the diabetes-induced rat groups (DC, DFO, and DLO) in terms of food and water consumption (p>0.05), whereas these groups were observed to consume significantly more food (p<0.001, p<0.001, and p=002, respectively) and water (p<0.001) compared to the control group. At the same time, the diabetes-induced rat groups (DC, DLO, and DFO) lost weight during the feeding period, whereas the rats in group C gained weight (Table 1).

**Fasting blood glucose**

**Figure 1:**
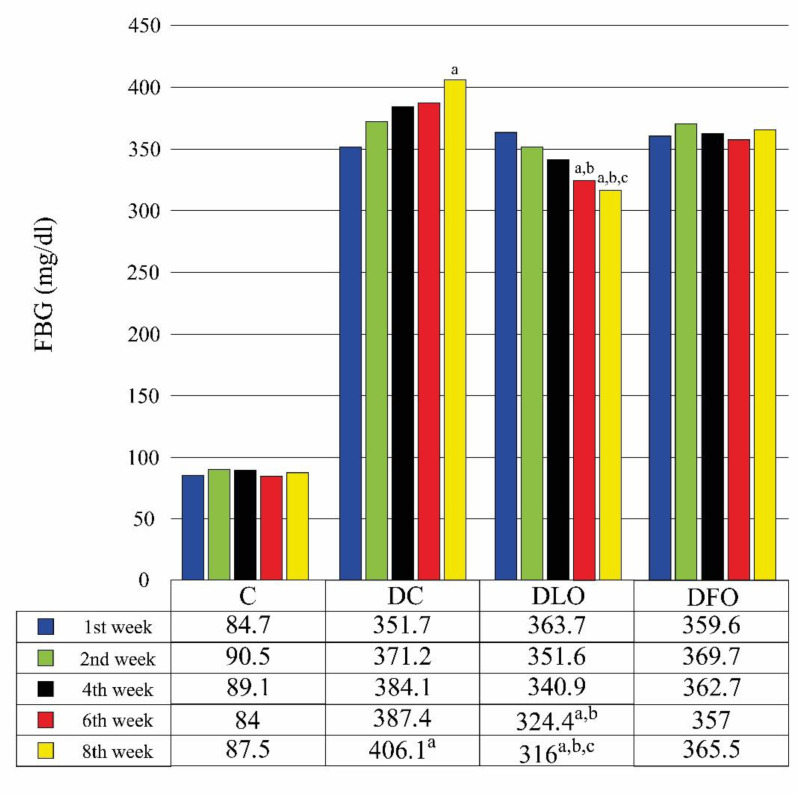
Groups’ mean fasting blood glucose values (mg/dl) measured during the experiment (mean, n=8)

^a ^Significantly different from the 1^st^ week. ^b ^Significantly different from the 2^nd^ week. ^c ^Significantly different from the 4^th^ week. ^a,b,c ^(p<0.05). C: Control; DC: Diabetic Control; DLO: Diabetic + Lyophilized Onion; DFO: Diabetic + Furnaced Onion.

FBG levels of groups are shown in Figure 1. FBG values of DC group increased in the weeks after STZ injection. While the 8th week value was significantly higher than the 1st week value in the DC group (p=0.025), there was no significant difference between the values measured in the other weeks in this group (p>0.05). 

**Table 1:** Groups’ live body weights at the beginning and end of the experiment, and their mean food and water consumption during the study period (mean ± SEM, n=8).

	**C**	**DC**	**DLO**	**DFO**
**AFI (g/day)**	17.9±0.4	22.0±0.6^a*^	20.9±0.5^a^	21.2±0.5^a*^
**AWI (mL/day)**	41.4±2.1	86±5.1^a*^	82±4.9^a*^	90.1±6.2^a*^
**IBW (g)**	299.7±3.3	303.5±2.7	308.8±3.4	301.0±2.8
**UBW (g)**	351.6±3.2	229.9±3.8^a*^	241.6±3.7^a*^	229.5±3.8^a*^

AFI; Average food intake, AWI; Average water intake, IBW; Initial body weight, UBW; Ultimate body weight. ^a^Significantly different from control group (p<0.05). ^a^(p<0.05), ^a*^(p<0.001). C: Control; DC: Diabetic Control; DLO: Diabetic + Lyophilized Onion; DFO: Diabetic + Furnaced Onion.

Among the diabetic groups, DLO group was the only group with a tendency to decrease in FBG values regularly. In the DLO group, the 6th week value was significantly lower than the 1st and 2nd week values (p=0.49, and p=0.027, respectively), and the 8th week value was significantly lower than the 1st, 2nd, and 4th week values (p=0.003, p=0.024, and p=0.01, respectively). There was no significant difference between the values measured at other weeks in the DFO group (p>0.05). Unlike the DLO group, there was a tendency to increase in FBG values of the DC group, but only a significant difference was observed between the 8th and 1st week values (p=0.025).

**Serum lipids**

The levels of TC, TG, HDL-C and LDL-C in the groups are shown in Table 2. No significant differences were found between the DFO and DC groups in terms of these values (p>0.05), whereas, the levels of TC, TG and LDL-C in the DLO group were significantly lower (p=0.016, p<0.001, and p=0.006, respectively) and HDL-C level of this group was significantly higher (p=0.049) than those of the DC group. Significant differences were also observed between DLO and DFO groups in terms of TC, TG and LDL-C levels and these values were found to be significantly lower in the DLO group (p=0.042, p<0.001, and p=0.006, respectively). At the same time, TC, TG, and LDL-C levels of DC group were found to be significantly lower than the levels of group C (p<0.001, p<0.001, and p=0.004, respectively), whereas no significant difference was found between these two groups in terms of HDL-C levels (p>0.05).

**Table 2:** Groups’ mean TC, TG, HDL-C and LDL-C values (mg/dl) at the end of the experiment (mean±SEM, n=8).

	**C**	**DC**	**DLO**	**DFO**
**TC **	78.9±4.2	122.5±7.3^a*^	89.6±5.8^b,c^	118.6±8.3^a^
**TG **	78.4±5.4	198.4±13.1^a*^	99.6±8.1^b*,c*^	185.4±15.2^a*^
**HDL-C **	33.2±2.9	24.9±2.1	35.9±2.4^b^	27.2±2.8
**LDL-C **	37.7±3.2	56.4±4.0^a^	38.2±2.8^b,c^	52.2±3.3^a^

TC; Total cholesterol, TG; Triglyceride, HDL-C; High density lipoprotein cholesterol, LDL-C; Low density lipoprotein cholesterol. Total Values are given as mean±SE (n=8). ^a ^Significantly different from group C. ^b ^Significantly different from group DC. ^c ^Significantly different from group DFO. ^a,b,c ^(p<0.05), ^a*,b*,c* ^(p<0.001). C: Control; DC: Diabetic Control; DLO: Diabetic + Lyophilized Onion; DFO: Diabetic + Furnaced Onion.

## Discussion

In the present study, it was concluded that there was a significant decrease in FBG values following treatment with lyophilized onion powder but no significant change was observed following the use of furnaced onion powder according to measurements done at different weeks. Differences were also found between the effects of lyophilized onion powder and furnaced onion powder on diabetic dyslipidemia and it was found that lyophilized onion powder had positive effects against diabetic dyslipidemia. Another finding of the study is that neither lyophilized nor furnaced onion powder could not adequately protect the live body weight of diabetic rats against proteolytic and lipolytic effects of DM.

Polyphagia and polydipsia are the most common signs and symptoms of DM (ADA, 2014 ▶). When the food and water consumption profiles of rat groups were examined in the present study (Table 1), the diabetes-induced rat groups (DC, DLO, and DFO) were observed to consume more food and water than the control group. At the same time, despite the increase in their food consumption, the diabetes-induced rat groups experienced weight loss in the 8-week feeding period. The lipolysis and gluconeogenesis occurring to meet cell energy needs in the presence of DM, cause weight loss (Quinn, 2002 ▶; Sindhu et al., 2004 ▶). In addition, loss of energy due to glucose excretion through urine is another cause of weight loss in diabetic patients. Studies reported weight loss in rats with diabetes induced using streptozotocin injection (Ahn et al., 2006 ▶; Pandit et al., 2010 ▶; Roghani and Baluchnejadmojarad, 2010 ▶; Zafar and Naqvi, 2010 ▶). In this regard, our results are consistent with the literature.

Onion provides a hypoglycemic effect by regulating the activities of certain enzymes involved in carbohydrate metabolism (hexokinase, glucose 6-phosphatase and hydroxy 3-methyl glutaryl coenzyme reductase), increasing insulin secretion and sensitivity, and enhancing NADP+ and NADPH activities, due to its content of organosulfur compounds and flavonoids such as quercetin (Akash et al., 2014 ▶). Quercetin also inhibits the enzyme α-glucosidase, thus prevents the formation of D-glucose from oligosaccharides and disaccharides, and delays the absorption of glucose from the intestine (Kim et al., 2011 ▶). In this study, it was found that the hypoglycemic activity of onion changed depending on the applied heat treatments. Lyophilized onion powder was effective in lowering plasma glucose levels but no statistically significant effect was observed for furnaced onion powder. Similar effects were observed in studies about the effects of lyophilized onion powder on FBG levels (Azuma et al., 2007 ▶; Bang et al., 2009 ▶; Yoshinari et al., 2012 ▶), which are consistent with the relevant results obtained in the present study. Studies evaluating the effect of onion powders dried by heat treatment, on FBG levels were not found in the literature. It is thought that the change in onion powders’ content of flavonoid and sulphurous compounds due to different heat treatments, may explain why the onion powders dried in lyophilizator and furnace did not have the same effect in rats. Indeed, it was reported that the amount of sulphurous compounds in the garlic with high content of sulphurous compounds, was greatly reduced by boiling the garlic at 90°C for 5 min (Beato et al., 2012 ▶). In another study, researchers observed that the amount of quercetin in onions decreased depending on the increased temperature grades (Sharma et al., 2015 ▶).

Diabetic dyslipidemia is one of the major complications of diabetes and occurs in diabetic patients due to insulin insufficiency or insensitivity (Tangvarasittichai, 2015 ▶). Besides hypercholesterolemia and hypertriglyceridemia, other characteristics of diabetic dyslipidemia are decrement of HDL-C concentration, and increment of LDL-C and VLDL concentrations (Tangvarasittichai, 2015 ▶). According to the results, the mean TG, TC and LDL-C levels of the DC and DFO groups were significantly higher than those of the C group, but no significant difference was found between the DC and DFO groups in terms of these parameters and HDL-C levels (Table 2). In addition, significant differences were observed between DLO and DFO groups in terms of TC, TG and LDL-C levels and it was found that lyophilized onion powder had positive effects against diabetic dyslipidemia. Studies reported that TC, TG and LDL-C levels are increased and HDL-C levels are decreased in the presence of diabetes (Smith and Lall, 2008 ▶; Wu and Parhofer, 2014 ▶). A study examining the effects of rat diets including onion powder on plasma lipid profile of diabetes-induced rats, found that lyophilized onion powder decreased LDL-C, TG and TC levels and increased HDL-C levels (Babu and Srinivasan, 1997 ▶). Another similar study determined that lyophilized onion powder decreased TG and TC levels (Bang et al., 2009 ▶). The results obtained in these studies are compatible with those of the present study. Similar results were obtained when quercetin and organosulfur compounds of onion, which were considered to produce hypolipidemic effects, were given separately to rats. When quercetin was separately given to diabetic rats, plasma TC and TG levels were observed to decrease significantly (Vessal et al., 2003 ▶). It was reported that when s-methyl cysteine, a sulphurous compound thought to play a role in producing hypolipidemic effect of onion, was separately given to rats fed with high fructose, LDL-C and TG levels decreased and HDL-C levels increased significantly (Senthilkumar et al., 2013). In addition, it was reported that these effects of onion and its sulphurous compounds on plasma lipid profile, were produced by inhibiting HMG-CoA reductase activities and decreasing intestinal cholesterol absorption (Hasimun et al., 2011 ▶). Moreover, the amount of cholesterol excretion through feces was found to be 3 times more in rats fed with rat diets including s-methyl cysteine (Hasimun et al., 2011 ▶). It is also believed that onion may protect against diabetic dyslipidemia by regulating lipoprotein lipase and hepatic lipase activities due to its insulinotropic effects. 

Studies evaluating the effect of onion powders dried by heat treatment on plasma lipid profile, were not found in the literature. It is thought that as opposed to the beneficial effects of lyophilized onion powder, the change in the content of flavonol and sulphurous compounds depending on the increased temperature values prevents furnace-dried onion powders from having a significant effect on plasma lipid profile of the diabetes-induced rats. Indeed, the similar effects observed when these useful compounds were administered alone and the change in flavonol and sulphurous compound proportions depending on the increased temperature values *in vitro* studies support this opinion.

As the main limitation of our study, we focused on the changes in the functional efficiency of onion by drying method in only two different heat treatments (+80°C and -76°C). However, it is important to determine the changes in the functional activity of onions due to different cooking methods (frying, boiling, baking, etc.) at different temperatures. In this way, studies can give an idea of the ideal consumption aspects for foods that are thought to have functional activity against diabetes. In addition, it may be useful to evaluate the efficacy in healthy rat models (positive control) in future studies.

The results suggest that lyophilized onion powder may be protective against hyperglycaemia and dyslipidemia arising from diabetes. However, the heat treatments applied to onion affect this protective role negatively. Consequently, it is an important issue to make patients acquire consumption habits with proper consumption forms of nutrients such as onions with nutritionally proven functional efficiency, in the medical nutrition therapy as one of the cornerstones of DM treatment. It is not possible to benefit from beneficial effects of these foods due to improper cooking and consumption patterns. Therefore, further clinical trial studies are needed to explain the effectiveness of different consumption forms of other foods that are thought to have a functional effect against DM.
